# Translating Translation: Lessons from Zotatifin’s Clinical Development and the Case for Trials in Lymphoma

**DOI:** 10.1007/s11912-026-01848-6

**Published:** 2026-10-28

**Authors:** Paola Manara, Jonathan H. Schatz

**Affiliations:** 1 Division of Hematology, Department of Medicine, University of Miami Miller School of Medicine, Miami, Florida USA; 2 Sylvester Comprehensive Cancer Center, Miami, Florida USA

**Keywords:** eIF4A1, Rocaglates, Zotatifin, Cap-dependent translation, DLBCL, Ferroptosis

## Abstract

**Purpose of Review:**

The eIF4F complex initiates cap-dependent translation and sits downstream of several oncogenic signaling pathways. Its enzymatic core, the RNA helicase eIF4A1, is of interest as a drug target because inhibiting it reduces the synthesis of many oncoproteins at once. We review the basis for targeting eIF4A1, the clinical experience with drugs directed at the eIF4F axis, and the disease settings that should be prioritized next.

**Recent Findings:**

Rocaglates, the best-studied eIF4A1 inhibitors, clamp eIF4A1 onto polypurine sequences in 5' untranslated regions and deplete the corresponding mRNAs from the translating pool. The synthetic rocaglate zotatifin (eFT226) is the only compound of this class tested in patients, and its trials were limited to solid tumors before its developer wound down in 2024. In B-cell malignancies, eIF4A1 inhibition suppresses NRF2 translation. NRF2 loss alone does not cause ferroptosis, but it lowers the threshold for ferroptosis inducers and increases killing by CD19-directed chimeric antigen receptor (CAR) T cells in diffuse large B-cell lymphoma (DLBCL) models. Other eIF4A-directed compounds, including pateamine A derivatives and hippuristanol, remain preclinical. Zotatifin is now under new ownership, and trials in lymphoma and other hematologic malignancies are anticipated.

**Summary:**

The preclinical evidence for eIF4A1 inhibition is strongest in B-cell malignancies, particularly DLBCL, but no trial has tested it in this setting. We argue that lymphoma should be prioritized in future trials, and that combinations with ferroptosis inducers and with CAR T-cell therapy should be evaluated early.

## Introduction

The eIF4F complex assembles at the 5′ cap of mRNA and is where several oncogenic signaling pathways converge. Inhibitors of cap-dependent initiation were first identified through phenotypic screening [1]. Pelletier and colleagues used a bicistronic in vitro translation reporter to find natural compounds that block cap-dependent translation while sparing IRES-mediated translation. This identified silvestrol, a rocaglate from *Aglaia* plant species, as a selective inhibitor of eIF4A-dependent initiation, and showed that it resensitized tumor cells to chemotherapy in a mouse lymphoma model [2]. The approach was subsequently extended to human B-cell lymphoma, where inhibiting cap-dependent translation was shown to block the survival signals converging from AKT and PIM kinases, producing activity that kinase inhibitors alone did not achieve [3].

Targeting individual oncoproteins is repeatedly undermined by redundancy in tumor signaling, where resistance emerges through parallel pathway activation or secondary mutation. Inhibiting translation instead cuts the supply of several oncoproteins at once. Because this acts at the level of translation rather than signal transduction, it does not depend on which upstream kinase pathway is active. Selectivity comes from the mRNAs themselves rather than from any mutation in the translation machinery [4, 5]. eIF4A1 is a DEAD-box RNA helicase that unwinds secondary structure in 5′ untranslated regions to permit ribosome scanning [6]. Transcripts encoding oncoproteins are enriched for structured, polypurine-rich 5′ UTRs and depend heavily on this activity, whereas most housekeeping mRNAs have short, unstructured 5′ UTRs and tolerate reduced eIF4A1 function [4, 7]. This difference is the basis of the therapeutic window.

B-cell malignancies emerged from these early studies as a sensitive tumor type, and the finding has been reproduced across independent laboratories and compound classes [8, 9]. Yet despite more than two decades of preclinical work, and a longer history of clinical attempts to target cap-dependent translation at several nodes of the eIF4F axis, only one eIF4A1 inhibitor has reached patients. Zotatifin was tested in solid tumors rather than in the hematologic malignancies where the evidence is strongest. The 2024 wind-down of its developer, eFFECTOR Therapeutics, following disappointing mid-stage results, is a reason to reconsider where the field stands. Here we examine eIF4A1 as a drug target, review the clinical experience with cap-dependent translation inhibitors, analyze the available results from zotatifin’s program, and set out a path forward in B-cell lymphoma.

## eIF4A1 as a Drug Target in Cancer

### The eIF4F Complex and Cap-Dependent Translation

**Fig. 1:**
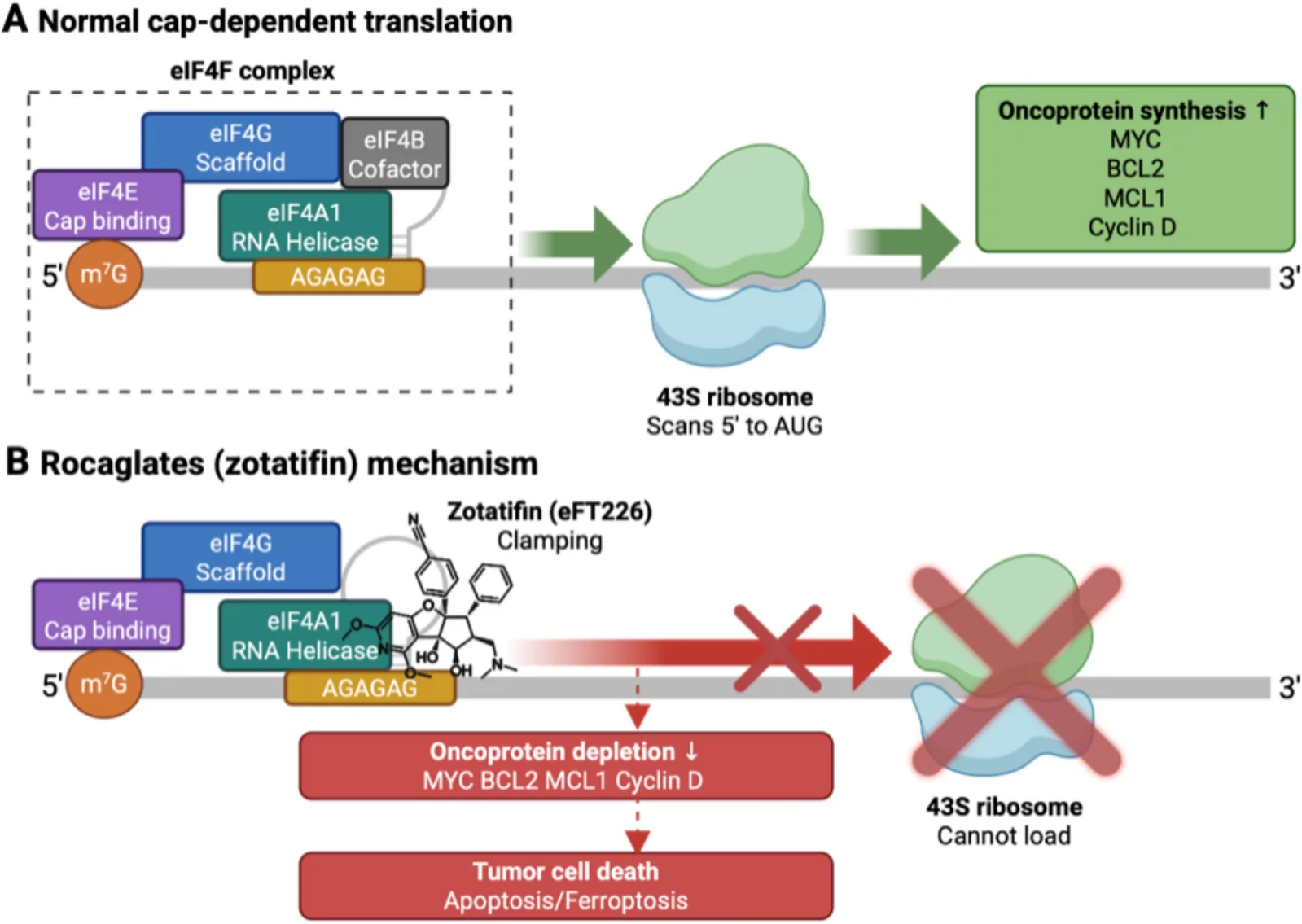
The eIF4F complex and the mechanism of rocaglate/zotatifin-mediated translation inhibition. (**A**) Under normal conditions, the eIF4F complex assembles at the 5′ m^7^G cap of mRNA, with eIF4E binding the cap, eIF4G acting as a scaffold, and the ATP-dependent RNA helicase eIF4A1 (assisted by eIF4B) unwinding secondary structure in the 5′ untranslated region to permit 43S ribosomal scanning and synthesis of oncoproteins such as MYC, MCL1, BCL2, and cyclin D1. (**B**) Rocaglates, including the synthetic derivative zotatifin (eFT226), act as molecular glues that clamp eIF4A1 onto polypurine sequences in the 5′ UTR, forming a ternary drug–eIF4A1–RNA complex that stalls ribosome scanning, depletes oncoprotein-encoding mRNAs from the translationally active pool, and drives tumor cell death through apoptosis and ferroptosis. Abbreviations: 5′ UTR, 5′ untranslated region; eIF, eukaryotic initiation factor; m^7^G, 7-methylguanosine. Created in BioRender. Manara, P. (2026) https://BioRender.com/5u9jwe6

Translation of most eukaryotic mRNAs begins when eIF4E, the cap-binding subunit of eIF4F, recognizes the 5′ m^7^G cap. This nucleates assembly of the full complex, in which eIF4G bridges eIF4E to eIF4A1 and the 43S pre-initiation complex [10, 11]. eIF4A1 then acts as an ATP-dependent helicase, assisted by eIF4B and eIF4H, unwinding secondary structure in the 5′ UTR so the ribosome can scan toward the start codon (Fig. 1). This step is rate-limiting for initiation and is regulated by oncogenic signaling [12].

mTORC1 controls eIF4F assembly by phosphorylating 4E-binding proteins (4E-BPs), which sequester eIF4E when unphosphorylated [13]. Downstream of PI3K-AKT and RAS-MAPK signaling, MNK1/2 kinases phosphorylate eIF4E at Ser209 [14]. The tumor suppressor PDCD4 restrains eIF4A1, and its loss in cancer raises eIF4A1 activity further [15]. In most malignancies eIF4F is therefore constitutively active, which is both a dependency and a vulnerability.

### Structural Basis for mRNA Selectivity

Not all mRNAs depend equally on eIF4A1. Transcripts encoding MYC, MCL1, cyclin D1, BCL2, and CDK4/6 carry GC-rich 5′ UTRs with G-quadruplex structures and polypurine stretches that require active unwinding [4]. Most housekeeping transcripts have short, unstructured 5′ UTRs and can be translated with residual eIF4A1 activity or through cap-independent mechanisms [7].

The practical consequence is that partial inhibition of eIF4A1 suppresses the oncoprotein-encoding subset disproportionately, without shutting down global protein synthesis. Because this occurs downstream of the kinases that activate eIF4F components, the same breadth of suppression is difficult to achieve with an upstream signaling inhibitor [2, 3].

### The Rocaglate Mechanism

Rocaglates, natural compounds from *Aglaia* species and their synthetic derivatives, are the most studied class of eIF4A1 inhibitors [16]. They do not block the enzyme competitively. Instead they act as molecular glues, locking eIF4A1 onto polypurine RNA [5]. The resulting drug-eIF4A1-RNA complex acts as a roadblock, stalling 40S subunits in the 5′ UTR and preventing the ribosome from reaching the start codon [17].

The clamping is sequence-selective. Rocaglates preferentially trap eIF4A1 on AG-rich sequences, which are over-represented in oncoprotein 5′ UTRs, and have little effect on eIF4A1 engagement with simple mRNAs [18]. Structural work shows that rocaglates also clamp the paralog eIF4A2 and the DEAD-box helicase DDX3, with drug- and sequence-specific differences across these targets [19]. This gain-of-function pharmacology sets rocaglates apart from other translation inhibitors and accounts for their potency, with effective concentrations in the low nanomolar range in sensitive cancer cell lines.

### Oncoproteins Suppressed by Rocaglates

Rocaglate treatment rapidly reduces MYC in B-cell malignancies, consistent with its structured 5′ UTR and short protein half-life [8]. MCL1, an anti-apoptotic factor in lymphoma and multiple myeloma, also declines quickly, and combining eIF4A inhibition with BH3 mimetics is synergistic in acute myeloid leukemia [20]. Cyclin D1, CDK4, and CDK6 show strong translational dependence on eIF4A1 [9]. BCL6, the master transcription factor of the germinal center reaction and a defining oncogene of GCB-DLBCL, is likewise suppressed [8].

Suppressing this set of short-lived oncoproteins at once is most useful in tumors driven by several pathways at the same time, where blocking any single node leaves the others intact. In animal studies, partial translation inhibition was tolerated by normal tissues.

## Clinical Experience Targeting Cap-Dependent Translation

**Fig. 2:**
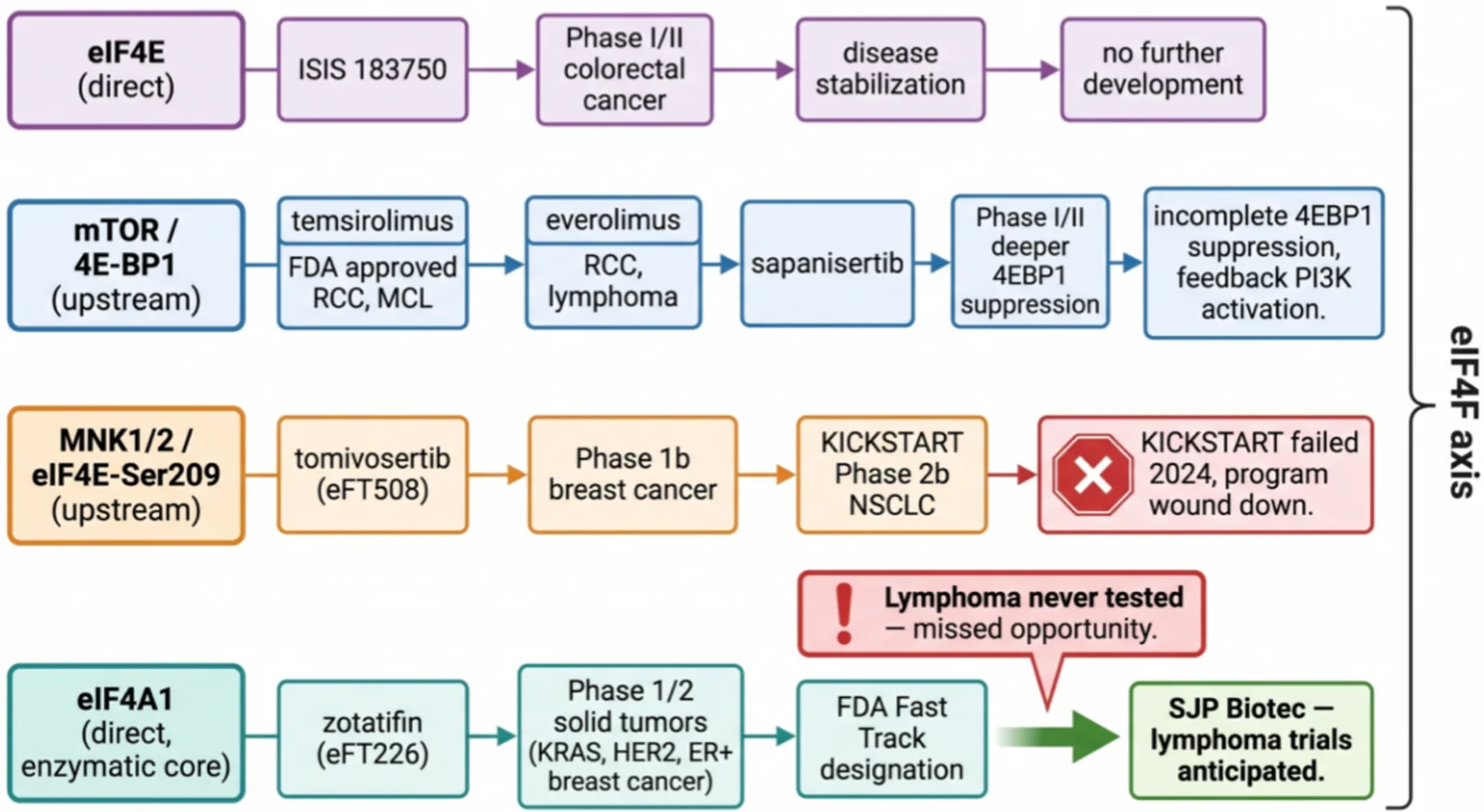
Clinical landscape of agents targeting cap-dependent translation across the eIF4F axis. Clinical efforts have targeted four distinct nodes of the cap-dependent translation apparatus: direct inhibition of eIF4E (antisense oligonucleotide ISIS 183750); upstream modulation of eIF4F assembly through mTORC1 (rapalogs such as temsirolimus and everolimus, and ATP-competitive inhibitors such as sapanisertib); inhibition of MNK1/2-mediated eIF4E phosphorylation (tomivosertib); and direct inhibition of the enzymatic core eIF4A1 (zotatifin). Across these programs, target engagement and biological proof of concept were demonstrable, but registration-quality efficacy was not achieved, and none was designed and powered for the B-cell malignancies where the preclinical rationale is strongest. Abbreviations: ATP, adenosine triphosphate; eIF, eukaryotic initiation factor; MCL, mantle cell lymphoma; MNK, MAPK-interacting kinase; mTORC1, mechanistic target of rapamycin complex 1; NSCLC, non-small cell lung cancer; RCC, renal cell carcinoma. Created in BioRender. Manara, P. (2026) https://BioRender.com/n1pw6b0

Clinical efforts have targeted four nodes of the eIF4F axis: eIF4E directly, mTOR upstream of eIF4F assembly, MNK-mediated eIF4E phosphorylation, and eIF4A1 itself (Fig. 2).

### Targeting eIF4E

eIF4E was among the first initiation factors recognized as an oncogene. It is overexpressed in roughly 30% of human cancers, and forced overexpression transforms cells in model systems [21–23]. ISIS 183750, a second-generation antisense oligonucleotide against eIF4E mRNA, was tested in a Phase I/II trial in refractory solid tumors, with expansion into irinotecan-refractory colorectal cancer in combination with irinotecan [24]. There were no dose-limiting toxicities. Target engagement was confirmed by reduced eIF4E mRNA in blood and matched tumor biopsies in most treated patients, and 7 of 15 evaluable colorectal cancer patients had disease stabilization, with a median duration of disease control of 22.1 weeks. No objective responses occurred, and no further development of eIF4E-directed antisense therapy has been reported.

### Targeting Upstream of eIF4F: Rapalogs and Active-Site mTOR Inhibitors

mTORC1 controls eIF4F assembly through 4E-BP and S6 kinase phosphorylation, and its inhibition is the most clinically validated way to disrupt cap-dependent translation indirectly. Temsirolimus was approved for renal cell carcinoma (RCC) in 2007, and a Phase III trial in relapsed mantle cell lymphoma (MCL) showed a 22% overall response rate against investigator’s choice, with superior progression-free survival, supporting approval in MCL in Europe [25]. Everolimus produced responses in relapsed aggressive lymphoma, and T-cell lymphoma in Phase II trials, with overall response rates of 38–44% in some subtypes [26, 27].

Two limitations emerged. Allosteric mTORC1 inhibitors suppress 4EBP1 phosphorylation incompletely, and they relieve S6 kinase-mediated feedback on PI3K-AKT, which partly restores cap-dependent translation [28]. Active-site, ATP-competitive mTOR inhibitors including sapanisertib, CC-214, and AZD8055 were developed to block both mTORC1 substrates and mTORC2. In lymphoma models they produced more complete 4EBP1 dephosphorylation and better anti-tumor activity than rapalogs [29]. In Phase I/II trials in solid tumors and hematologic malignancies they were tolerable and showed deeper target engagement, but none reached registration [29–32]. Loss of 4EBP1 expression in some lymphoma subsets is one resistance mechanism affecting this whole approach.

### Targeting MNK1/2: eIF4E Phosphorylation Inhibitors

MNK1/2 phosphorylate eIF4E at Ser209, which promotes translation of a subset of oncoproteins. Tomivosertib (eFT508), developed by eFFECTOR Therapeutics, was the most clinically tested compound in this class. A Phase 1b trial combining tomivosertib with paclitaxel in refractory metastatic breast cancer showed acceptable tolerability, no pharmacokinetic interaction, and reduced phospho-eIF4E (Ser209) in tumor biopsies [33]. The randomized Phase 2b KICKSTART trial then tested tomivosertib with pembrolizumab in frontline metastatic NSCLC, on the hypothesis that lowering eIF4E phosphorylation would de-repress PD-L1 translation and improve checkpoint inhibitor sensitivity. KICKSTART missed its primary progression-free survival endpoint in early 2024, which ended the eFFECTOR program [34]. An investigator-sponsored study of tomivosertib in acute myeloid leukemia, based on suppression of MCL1 and BCL2 translation, continued independently.

### Targeting eIF4A1: Zotatifin

Zotatifin (eFT226) is the first and so far only direct eIF4A1 inhibitor to enter clinical evaluation. Its Phase 1/2 trial (NCT04092673) enrolled patients with solid tumors carrying KRAS mutations, HER2 amplification, ERBB3 alterations, or FGFR1/2 alterations, with a later expansion cohort in estrogen receptor-positive (ER+) breast cancer [35, 36]. At the recommended Phase 2 dose, toxicities were mainly fatigue, anemia, nausea, and diarrhea, and were mostly mild and reversible. Activity appeared in the ER+ breast cancer cohort, with partial responses in patients with cyclin D1 amplification or ESR1 mutations treated with zotatifin plus fulvestrant, or with zotatifin, fulvestrant, and abemaciclib [37]. The program received FDA Fast Track designation in ER+/HER2−metastatic breast cancer and was moving toward a randomized registrational trial when eFFECTOR’s wind-down, triggered by the KICKSTART failure, ended development.

Across all four nodes, target engagement was demonstrable but efficacy sufficient for registration was not achieved in any indication, and none of these programs was designed or powered for hematologic malignancies.

## Zotatifin: Preclinical Strength and Missed Opportunities

### Preclinical Validation in B-Cell Malignancies

The preclinical foundation for eIF4A1 inhibition was built in B-cell malignancies, in studies designed to find compounds active against the translational dependencies of lymphoma cells [2, 3]. Independent work then confirmed that rocaglates, including zotatifin, are active at nanomolar concentrations against DLBCL and mantle cell lymphoma cell lines and in patient-derived xenografts [8, 9]. Silvestrol showed B-cell selectivity in primary CLL and B-ALL in vitro and in mouse xenografts [38]. Sensitivity was mapped specifically to PIM-driven ABC-DLBCL, linking eIF4A1 inhibition to cap-dependent translation downstream of PIM [39]. Proteomic characterization of the rocaglate translatome by TMT-pSILAC defined the basis of this selectivity and identified GEF-H1/RHOA/JNK as an additional cytotoxic mechanism [40].

### Zotatifin’s Clinical Development: Solid Tumors

Zotatifin was tested exclusively in solid tumors, in NSCLC and breast cancer. Three features of these settings make them less favorable for eIF4A1 inhibition than B-cell lymphoma.

First, the oncogenic drivers are not uniformly translationally regulated. KRAS-mutant NSCLC activates several parallel pathways, including MEK/ERK, PI3K/AKT, and RAL, that do not all depend on eIF4A1-sensitive oncoproteins for survival, and KRAS itself has a relatively simple 5′ UTR compared with MYC and MCL1 [9].

Second, solid tumors are harder to expose to drug at therapeutically relevant concentrations than hematologic malignancies, where tumor cells circulate in blood or sit in lymphoid organs with good drug penetration.

Third, the preclinical literature had already identified B-cell lymphomas as the tumor type with the strongest eIF4A1 dependence. Germinal center B cells and their malignant counterparts sustain very high rates of protein synthesis to support clonal expansion and immunoglobulin production, which makes them sensitive to even partial translation inhibition.

Testing in larger solid tumor markets therefore left aside indications where an unambiguous efficacy signal was more likely. On the evidence set out below, a Phase 2 study in relapsed or refractory DLBCL, where options after CAR T-cell failure are limited, might have provided the proof of concept and the path to approval that solid tumor activity at Phase 1/2 doses did not.

## The Case for B-Cell Lymphoma as the Optimal Disease Area

### Oncogenic Architecture of DLBCL

Diffuse large B-cell lymphoma (DLBCL) is the most common aggressive non-Hodgkin lymphoma and accounts for 25–30% of lymphoma diagnoses [41]. Up to 40% of patients relapse after R-CHOP, and relapsed or refractory (R/R) disease carries a poor prognosis. CD19-directed CAR T-cell therapy (CAR-19) produces durable remissions in 30–40% of R/R patients, but most eventually progress with few options remaining [42, 43].

**Fig. 3:**
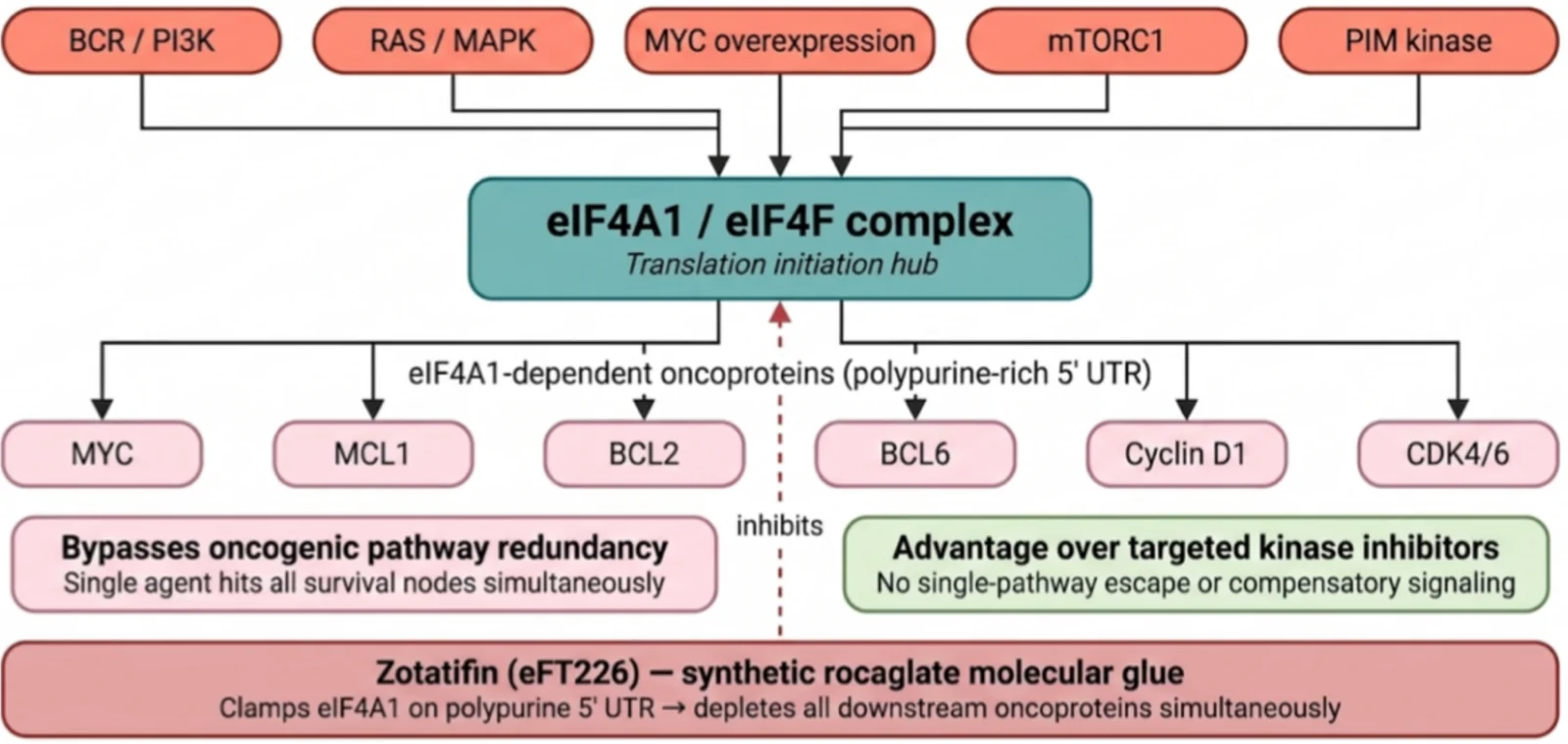
Oncogenic signaling convergence on eIF4A1 and the rationale for targeting translation in DLBCL. Multiple oncogenic drivers active in diffuse large B-cell lymphoma—including BCR/PI3K, RAS/MAPK, MYC overexpression, mTORC1, and PIM kinase signaling—converge on the activation of the eIF4F complex and eIF4A1-dependent translation. Because eIF4A1-dependent transcripts include the key oncoproteins that define aggressive B-cell lymphoma biology (MYC, MCL1, BCL2, BCL6, cyclin D1, and CDK4/6), all of which bear polypurine-rich 5′ UTRs, zotatifin simultaneously depletes this entire set. This bypasses the pathway redundancy and compensatory signaling that undermine single-target kinase inhibitors. Abbreviations: BCR, B-cell receptor; CDK, cyclin-dependent kinase; DLBCL, diffuse large B-cell lymphoma; eIF, eukaryotic initiation factor; MAPK, mitogen-activated protein kinase; mTORC1, mechanistic target of rapamycin complex 1; PI3K, phosphoinositide 3-kinase; 5′ UTR, 5′ untranslated region. Created in BioRender. Manara, P. (2026) https://BioRender.com/2a3ce8g

The drivers of DLBCL map onto the mechanism of eIF4A1 inhibition (Fig. 3). MYC, overexpressed through translocation, amplification, or transcriptional hyperactivation in a substantial fraction of cases, carries stable G-quadruplex structures in its 5′ UTR and is among the most translationally sensitive oncoproteins [4]. BCL6, the master transcription factor of the germinal center reaction and a survival factor in GCB-DLBCL, is regulated the same way [8]. MCL1, which confers resistance to apoptosis-targeted therapies, is strongly suppressed by rocaglate treatment [9]. AKT and PIM both converge on eIF4F to drive cap-dependent translation in DLBCL, and eIF4A1 inhibition acts downstream of both [3].

### Ferroptosis Sensitization: A Novel Therapeutic Dimension

NRF2 was first identified as a mediator of rocaglate resistance in a genome-wide CRISPR/Cas9 screen in murine pro-B cells [45], though the mechanism was not established. Work from our laboratory showed that zotatifin suppresses NRF2 at the translational level [44]. NRF2 is the master transcriptional regulator of the antioxidant response, and its loss lowers the threshold for ferroptosis, the iron-dependent cell death pathway driven by lipid peroxidation [46].

NRF2 mRNA carries multiple AG-rich sequences in its 5′ UTR that act as rocaglate clamping sites, making it a direct translational target of zotatifin. Rocaglate exposure also triggers a transcriptional stress response that is largely uncoupled from protein output, because the induced transcripts themselves require cap-dependent initiation. A small subset of ferroptosis-protective factors escapes this block and accumulates, most notably SLC3A2, the heavy-chain partner of system Xc−. As a result, zotatifin alone transiently raises glutathione rather than depleting it. This compensation does not substitute for the lost NRF2 program, and it is the arm that pharmacologic ferroptosis inducers dismantle, which explains the magnitude of the synergy [44].

NRF2 loss alone was not sufficient to induce ferroptosis, but it sensitized DLBCL cells to ferroptosis inducers including erastin analogs, RSL3, and imidazole ketone erastin (IKE) [44]. The combination of zotatifin and IKE controlled GCB-DLBCL patient-derived xenografts better than either agent alone [44]. The same mechanism was reported independently in osteosarcoma, where eIF4A1 inhibitors blocked NRF2 synthesis and prevented pulmonary metastasis [47].

**Fig. 4:**
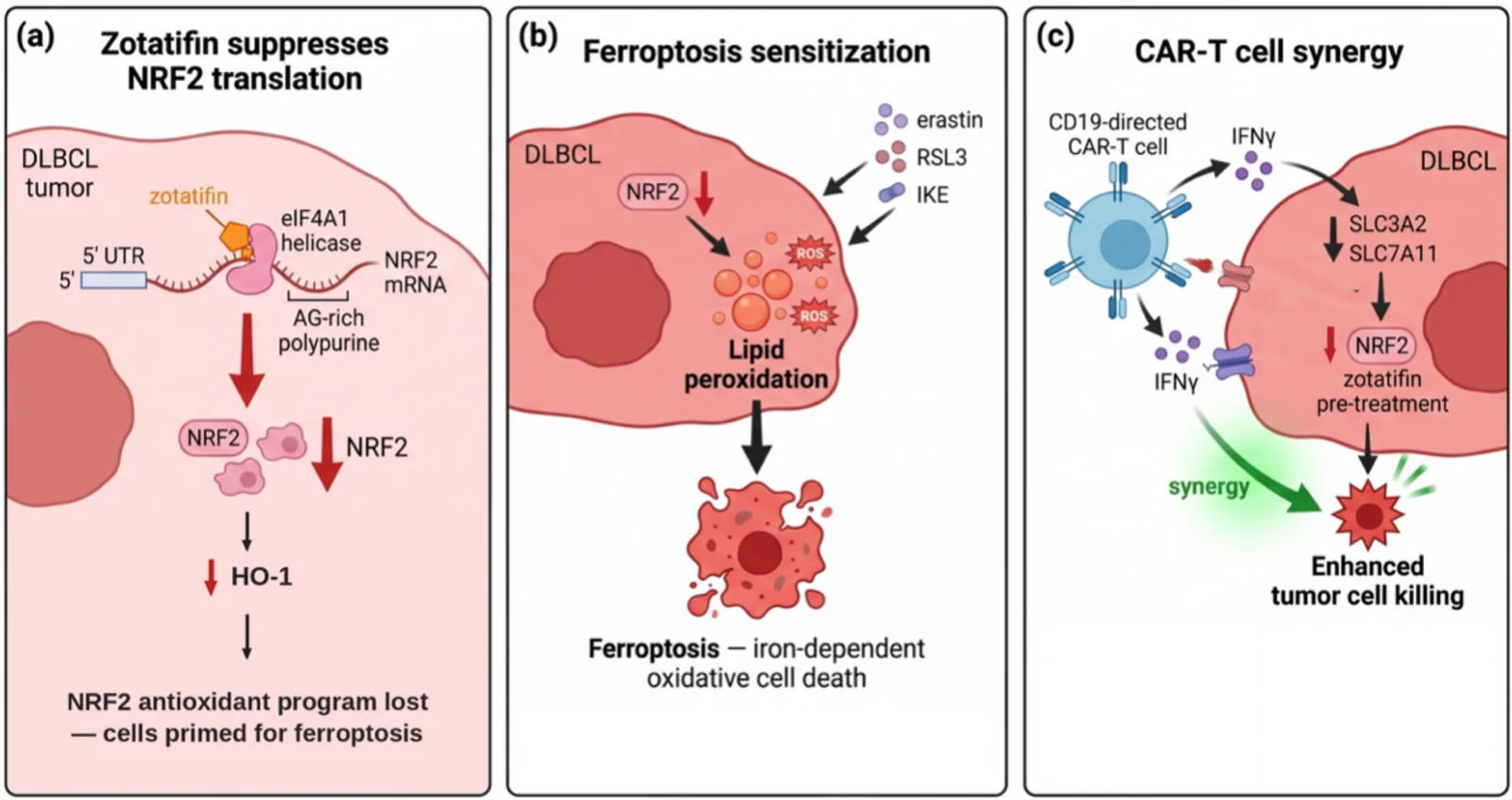
Translational suppression of NRF2 by zotatifin sensitizes DLBCL to ferroptosis and enhances CAR T-cell cytotoxicity. NRF2, the master transcriptional regulator of the antioxidant response, harbors AG-rich polypurine sequences in its 5′ UTR that render its translation dependent on eIF4A1 and therefore sensitive to rocaglate clamping. (**a**) Zotatifin blocks NRF2 translation, with parallel loss of the NRF2 target HO-1. NRF2 loss alone is insufficient to trigger ferroptosis; it lowers the antioxidant threshold and primes cells for death under additional oxidative stress. (**b**) Pharmacologic ferroptosis inducers (erastin, RSL3, and imidazole ketone erastin) supply that second hit, collapsing glutathione production and driving lipid peroxidation and ferroptotic death; the zotatifin–IKE combination controlled germinal-center DLBCL patient-derived xenografts in vivo. (**c**) Because CD8+ and CAR T cells promote ferroptosis in target cells through IFNγ-mediated downregulation of system Xc− (SLC3A2 and SLC7A11), zotatifin pre-treatment enhances CD19-directed CAR T-cell killing. A pre-treatment and washout strategy circumvents zotatifin’s direct toxicity to CAR T-cell products, while engineering the rocaglate-resistant eIF4A1-F163L mutation into CAR T cells could permit concurrent drug and cell therapy. Abbreviations: CAR, chimeric antigen receptor; DLBCL, diffuse large B-cell lymphoma; eIF4A1, eukaryotic initiation factor 4A1; HO-1, heme oxygenase 1; IFNγ, interferon gamma; IKE, imidazole ketone erastin; NRF2, nuclear factor erythroid 2-related factor 2; ROS, reactive oxygen species; 5′ UTR, 5′ untranslated region. Created in BioRender. Manara, P. (2026) https://BioRender.com/b8gn8my

This sensitization extends to immunotherapy. CD8+ cytotoxic T cells, including CAR T cells, kill in part through IFNγ, which downregulates the system Xc− components SLC3A2 and SLC7A11 and promotes ferroptosis in target cells [48]. Zotatifin also synergizes with IFNγ in DLBCL cell lines, consistent with rocaglate-IFNγ synergy reported in triple-negative breast cancer [44, 49]. Zotatifin is directly cytotoxic to CAR T-cell products, so the two cannot be given together. A pre-treatment and washout strategy enhanced CD19-directed CAR T-cell killing across several cell line models and CAR construct designs, particularly at low effector-to-target ratios, and extended survival in a syngeneic in vivo model, though transiently because of the drug’s half-life (Fig. 4) [44]. Engineering the rocaglate-resistant eIF4A1-F163L mutation into CAR T cells by CRISPR would allow concurrent drug and cell therapy without effector toxicity.

### Additional B-Cell Malignancy Indications

The rationale extends beyond DLBCL. Mantle cell lymphoma, driven by t(11;14) and cyclin D1 overexpression, is highly sensitive because the cyclin D1 5′ UTR is heavily structured [9, 50]. Multiple myeloma, with its high rate of immunoglobulin synthesis, is similarly vulnerable [51]. Burkitt lymphoma and double-hit lymphomas with concurrent MYC and BCL2 or BCL6 rearrangements are strong candidates because the drivers are translationally regulated [9, 45]. Chronic lymphocytic leukemia has shown preclinical sensitivity, particularly combined with BCL2 inhibitors [52].

## Zotatifin’s Future and Alternative eIF4A-Directed Candidates for Clinical Development

The wind-down of eFFECTOR slowed development of one compound rather than closing the field. The eFFECTOR compounds are now held by SJP Biotec (https://www.sjpbiotec.com/), which is continuing zotatifin’s clinical development, including trials in lymphoma and other hematologic malignancies and an orally available formulation. Several other eIF4A1 inhibitors identified over the past two decades also have properties suited to clinical development.

### Natural Rocaglates: Rocaglamide A and Silvestrol, and the Synthetic Derivative CR-1-31B

Rocaglamide A (RocA) is the prototypical natural rocaglate, active at nanomolar concentrations in B-cell malignancy models through the same RNA-clamping mechanism as zotatifin [5, 18]. Its limitation is pharmacokinetic: RocA is a P-glycoprotein (Pgp) substrate, which reduces bioavailability and intracellular concentrations in Pgp-expressing tumors [53, 54]. A forward genetic ORFeome screen identified FOXP3 and NR1I3, both ABCB1/Pgp regulators, as modifiers of rocaglate response. Given RocA’s potency in B-lymphocyte models, these limitations are worth revisiting with current formulation approaches.

Silvestrol carries a dioxanyloxy substituent that extends its RNA-binding beyond the polypurine preference of simpler rocaglates [55]. Crystallography shows it engages a broader range of RNA sequences, which may widen the set of suppressed targets. Silvestrol is active in CLL, MCL, and DLBCL models but, like RocA, is subject to Pgp-mediated efflux, a limitation addressed in related scaffolds by chemical modification. CR-1-31B, a synthetic rocaglate, suppressed MYC, MCL1, and other oncoproteins in B-cell malignancy cell lines at nanomolar concentrations [8]. It was never taken into humans, but its structural data have informed the design of derivatives with better drug-like properties.

### Pateamine A and Derivatives

Pateamine A (PatA) is a macrolide from the marine sponge *Mycale sp*. that inhibits eIF4A1 by a clamping mechanism similar to rocaglates, promoting eIF4A1-RNA dimerization and blocking ribosome recruitment [56]. Des-methyl, des-amino pateamine A (DMDA-PatA) is a synthetic analog engineered to remove the Pgp substrate recognition that limits natural PatA [56]. It retains eIF4A1-inhibitory activity while avoiding Pgp-mediated efflux, which matters for tumors that use Pgp as a resistance mechanism. This scaffold is chemically distinct from the rocaglates and could be optimized for B-cell malignancies.

### Hippuristanol

Hippuristanol, from the coral *Isis hippuris*, inhibits eIF4A1 by a different mechanism [57–59]. Rather than clamping eIF4A1 onto RNA, it binds the C-terminal domain allosterically and prevents RNA binding altogether. Because it acts at a different site, it is not subject to the same resistance mechanisms, including Pgp-mediated efflux [59]. Hippuristanol is active in B-cell malignancy models and synergizes with ferroptosis inducers, though less strongly than zotatifin, consistent with a loss-of-function mechanism being less potent than clamping at equivalent concentrations [44]. It remains a candidate for tumors that develop resistance to rocaglates.

### Amidino-Rocaglates and Next-Generation Scaffolds

Amidino-rocaglates retain eIF4A1-inhibitory activity through RNA clamping with improved selectivity and pharmacokinetics [60]. Virtual screening of the RocA: eIF4A1:RNA complex identified RBF197, RBF203, and RBF208, which bind the rocaglate pocket while offering structural diversity that may address the pharmacokinetic and resistance limits of earlier compounds [61]. Acyl sulfamide-modified rocaglates are selectively active against glioblastoma stem cells, showing that chemical modification can tune tissue selectivity within the class [62]. For B-cell malignancies, optimizing synthetic rocaglates for Pgp-insensitivity, oral bioavailability, and a defined eIF4A1 versus DDX3 engagement ratio is a tractable chemistry program that could produce a candidate with better properties than zotatifin.

## Conclusions and Future Directions

The wind-down of zotatifin’s original developer set back clinical progress, but the biology behind the target has not changed. The founding screens [1, 2], and their extension to human disease [3] established a rationale in B-cell lymphoma that subsequent work has strengthened rather than weakened. Ferroptosis sensitization, NRF2 suppression, and synergy with CAR T-cell immunotherapy have since added dimensions beyond oncoprotein downregulation [44].

Three steps would move the field forward.

- First, a sponsor committed to hematologic oncology needs to take one of the available eIF4A1 inhibitors, whether zotatifin, a next-generation synthetic rocaglate, DMDA-PatA, or an optimized scaffold, into a trial designed for relapsed or refractory DLBCL and related aggressive B-cell malignancies. Patient selection should enrich for tumors with high eIF4A1-sensitive oncoprotein dependencies: DLBCL with MYC overexpression, double-hit or triple-hit lymphomas, or tumors with high NFE2L2/NRF2 expression.
- Second, combination with ferroptosis inducers should be investigated clinically, and clinically translatable inducers should be co-developed with eIF4A1 inhibitors from the start rather than added later. Toxicity will need attention, since in vivo studies showed initial hepatotoxicity at high combination doses that was manageable with dose adjustment [44].
- Third, combining eIF4A1 inhibition with CAR-19 therapy, potentially by engineering CAR T cells to carry the rocaglate-resistant eIF4A1-F163L mutation, would allow simultaneous drug and cell therapy. The engineering is demanding but feasible, and the unmet need after CAR-19 failure is substantial.

Zotatifin’s development strategy produced a setback, not a refutation. The case for eIF4A1 as a target in B-cell lymphoma is stronger now than when the first trials were designed.

## Key References

- Manara P, Newsam AD, Venkatarame Gowda Saralamma V, et al (2025) Blocking NRF2 Translation by Inhibition of Cap-Dependent Initiation Sensitizes Lymphoma Cells to Ferroptosis and CAR T-cell Immunotherapy. Cancer Res 85:3717–36.
  - ○ Shows that zotatifin suppresses NRF2 translation in DLBCL, priming cells for ferroptosis and enhancing CD19-directed CAR T-cell killing. Provides the direct preclinical basis for the lymphoma trials and combinations proposed in this review.
- Boyer JA, Rosen EY, Sharma M, et al (2025) eIF4A controls translation of estrogen receptor alpha and is a therapeutic target in advanced breast cancer. Proc Natl Acad Sci 122:e2424286122.
  - ○ Demonstrates that eIF4A governs ERα translation and that zotatifin produces clinical responses in ER+ breast cancer. The clearest patient-level evidence that eIF4A is a tractable drug target.
- Lizardo MM, Hughes C, Huang YZ, et al (2024) Pharmacologic inhibition of eIF4A blocks NRF2 synthesis to prevent osteosarcoma metastasis. Clin Cancer Res.
  - ○ Independent confirmation, in osteosarcoma, that eIF4A inhibition blocks NRF2 synthesis and metastasis. Shows the NRF2 mechanism is not restricted to lymphoma.
- Ferrario C, Mackey J, Gelmon KA, et al (2025) Phase Ib Pharmacodynamic Study of the MNK Inhibitor Tomivosertib (eFT508) Combined With Paclitaxel in Patients With Refractory Metastatic Breast Cancer. Clin Cancer Res 31:491–502.
  - ○ The most recent clinical pharmacodynamic data on an eIF4F-axis agent, showing target engagement in tumor biopsies without sufficient efficacy. Illustrates the gap between proof of mechanism and registration that motivates the review.
- Lee YS, Good JD, Awais N, et al (2026) Synergistic targeting of eIF4A-mediated translation initiation and apoptosis in acute myeloid leukemia. Blood Neoplasia 3:100202.
  - ○ Shows synergy between eIF4A inhibition and BH3 mimetics in AML through loss of MCL1 and apoptotic priming. Supports the combination strategy proposed for hematologic malignancies.
- Zhao N, Kabotyanski EB, Saltzman AB, et al (2023) Targeting eIF4A triggers an interferon response to synergize with chemotherapy and suppress triple-negative breast cancer. J Clin Invest 133:e172503.
  - ○ eIF4A inhibition triggers an interferon response that synergizes with chemotherapy. Provides the mechanistic basis for the eIF4A and IFNγ synergy, and the CAR T-cell synergy, discussed here.

## Data Availability

No datasets were generated or analysed during the current study.

## Declarations

### Figures

All figures (Figs. 1, 2, 3 and 4) are original and were created in BioRender under an academic publication license (publisher: Springer). The required BioRender citation for each figure is given at the end of its legend.
